# Supplementary material for: Unveiling the Bioactive Potential of the Invasive Jellyfish Phyllorhiza punctata Through Integrative Transcriptomic and Proteomic Analyses
Source: Biomolecules. 2025 Aug 4;15(8):1121. doi: 10.3390/biom15081121 (PMC12383608; doi:10.3390/biom15081121)
Supplement: Supplementary file 1 [file biomolecules-15-01121-s001.zip › Suplementary Material/FileS3/PEPFOLD4/172_10448/clusters.html]

 PEP-FOLD clusters


Download archive of all models  here   

|  |  |  |
| --- | --- | --- |
| # | file | sOPEP |
| # cluster 1 - Size: 82 |  |  |
|  | 172-model1 | -12.522 |
|  | 172-model1.1 | -12.522 |
|  | 172-model1.2 | -12.522 |
|  | 172-model1.3 | -12.522 |
|  | 172-model1.4 | -12.522 |
|  | 172-model1.5 | -12.522 |
|  | 172-model1.6 | -12.522 |
|  | 172-model1.7 | -12.522 |
|  | 172-model1.8 | -12.522 |
|  | 172-model1.9 | -12.522 |
|  | 172-model1.10 | -12.522 |
|  | 172-model1.11 | -12.522 |
|  | 172-model1.12 | -12.522 |
|  | 172-model1.13 | -12.522 |
|  | 172-model1.14 | -12.522 |
|  | 172-model1.15 | -12.522 |
|  | 172-model1.16 | -12.522 |
|  | 172-model1.17 | -12.522 |
|  | 172-model1.18 | -12.52 |
|  | 172-model1.19 | -12.52 |
|  | 172-model1.20 | -12.52 |
|  | 172-model1.21 | -12.52 |
|  | 172-model1.22 | -12.52 |
|  | 172-model1.23 | -12.52 |
|  | 172-model1.24 | -12.52 |
|  | 172-model1.25 | -12.52 |
|  | 172-model1.26 | -12.52 |
|  | 172-model1.27 | -12.52 |
|  | 172-model1.28 | -12.468 |
|  | 172-model1.29 | -12.468 |
|  | 172-model1.30 | -12.468 |
|  | 172-model1.31 | -12.468 |
|  | 172-model1.32 | -12.468 |
|  | 172-model1.33 | -12.468 |
|  | 172-model1.34 | -12.468 |
|  | 172-model1.35 | -12.468 |
|  | 172-model1.36 | -12.468 |
|  | 172-model1.37 | -12.468 |
|  | 172-model1.38 | -12.468 |
|  | 172-model1.39 | -12.468 |
|  | 172-model1.40 | -12.468 |
|  | 172-model1.41 | -12.468 |
|  | 172-model1.42 | -12.468 |
|  | 172-model1.43 | -12.468 |
|  | 172-model1.44 | -12.468 |
|  | 172-model1.45 | -12.4541 |
|  | 172-model1.46 | -12.4541 |
|  | 172-model1.47 | -12.4541 |
|  | 172-model1.48 | -12.4541 |
|  | 172-model1.49 | -12.4541 |
|  | 172-model1.50 | -12.4541 |
|  | 172-model1.51 | -12.4541 |
|  | 172-model1.52 | -12.4541 |
|  | 172-model1.53 | -12.4541 |
|  | 172-model1.54 | -12.4541 |
|  | 172-model1.55 | -12.4541 |
|  | 172-model1.56 | -12.4148 |
|  | 172-model1.57 | -12.4141 |
|  | 172-model1.58 | -12.4141 |
|  | 172-model1.59 | -12.4141 |
|  | 172-model1.60 | -12.4141 |
|  | 172-model1.61 | -12.4141 |
|  | 172-model1.62 | -12.4141 |
|  | 172-model1.63 | -12.4141 |
|  | 172-model1.64 | -12.4141 |
|  | 172-model1.65 | -12.4141 |
|  | 172-model1.66 | -12.4141 |
|  | 172-model1.67 | -12.4141 |
|  | 172-model1.68 | -12.3916 |
|  | 172-model1.69 | -12.3916 |
|  | 172-model1.70 | -12.3916 |
|  | 172-model1.71 | -12.3916 |
|  | 172-model1.72 | -12.3916 |
|  | 172-model1.73 | -12.3916 |
|  | 172-model1.74 | -12.3916 |
|  | 172-model1.75 | -12.3472 |
|  | 172-model1.76 | -12.3472 |
|  | 172-model1.77 | -12.3472 |
|  | 172-model1.78 | -12.3472 |
|  | 172-model1.79 | -12.3472 |
|  | 172-model1.80 | -12.3201 |
|  | 172-model1.81 | -12.2256 |
| # cluster 2 - Size: 1 |  |  |
|  | 172-model2 | -6.28255 |
| # cluster 3 - Size: 7 |  |  |
|  | 172-model3 | -5.62626 |
|  | 172-model3.1 | -5.41723 |
|  | 172-model3.2 | -5.30672 |
|  | 172-model3.3 | -5.23893 |
|  | 172-model3.4 | -4.9595 |
|  | 172-model3.5 | -4.94116 |
|  | 172-model3.6 | -4.83147 |
| # cluster 4 - Size: 5 |  |  |
|  | 172-model4 | -4.73799 |
|  | 172-model4.1 | -4.7034 |
|  | 172-model4.2 | -4.36393 |
|  | 172-model4.3 | -4.23491 |
|  | 172-model4.4 | -3.14811 |
| # cluster 5 - Size: 2 |  |  |
|  | 172-model5 | -2.69368 |
|  | 172-model5.1 | -2.68337 |
| # cluster 6 - Size: 1 |  |  |
|  | 172-model6 | -2.65136 |
| # cluster 7 - Size: 1 |  |  |
|  | 172-model7 | -2.64591 |
| # cluster 8 - Size: 1 |  |  |
|  | 172-model8 | -2.60267 |
|  |  |  |
